# Expression of Curli by *Escherichia coli* O157:H7 Strains Isolated from Patients during Outbreaks Is Different from Similar Strains Isolated from Leafy Green Production Environments

**DOI:** 10.3389/fcimb.2016.00189

**Published:** 2016-12-20

**Authors:** Subbarao V. Ravva, Chester Z. Sarreal, Michael B. Cooley

**Affiliations:** Produce Safety and Microbiology Research Unit, Western Regional Research Center, Agricultural Research Service, U.S. Department of AgricultureAlbany, CA, USA

**Keywords:** O157:H7, curli, *E. coli*, leafy greens, spinach, outbreaks, MLVA, phylogenetic analysis

## Abstract

We previously reported that the strains of *Escherichia coli* O157:H7 (EcO157) that survived longer in austere soil environment lacked expression of curli, a fitness trait linked with intestinal colonization. In addition, the proportion of curli-positive variants of EcO157 decreased with repeated soil exposure. Here we evaluated 84 and 176 clinical strains from outbreaks and sporadic infections in the US, plus 211 animal fecal and environmental strains for curli expression. These shiga-toxigenic strains were from 328 different genotypes, as characterized by multi-locus variable-number tandem-repeat analysis (MLVA). More than half of the fecal strains (human and animal) and a significant proportion of environmental isolates (82%) were found to lack curli expression. EcO157 strains from several outbreaks linked with the consumption of contaminated apple juice, produce, hamburgers, steak, and beef were also found to lack curli expression. Phylogenetic analysis of fecal strains indicates curli expression is distributed throughout the population. However, a significant proportion of animal fecal isolates (84%) gave no curli expression compared to human fecal isolates (58%). In addition, analysis of environmental isolates indicated nearly exclusive clustering of curli expression to a single branch of the minimal spanning tree. This indicates that curli expression depends primarily upon the type of environmental exposure and the isolation source, although genotypic differences also contribute to clonal variation in curli. Furthermore, curli-deficient phenotype appears to be a selective trait for survival of EcO157 in agricultural environments.

## Introduction

Shiga-toxigenic *Escherichia coli* O157:H7 (EcO157) is responsible for over 96,000 diarrheal illnesses and 3200 hospitalizations annually in the United States (Scallan et al., 2011). One-fourth of the EcO157 associated outbreaks during 1998 to 2008 were linked with the consumption of contaminated leafy vegetables, fruits and nuts (Gould et al., 2013). Major outbreaks associated with produce indicate that pre-harvest contamination has occurred often, so it is critical to identify sources of pathogens (Mandrell, 2009). EcO157 responsible for 200 infections in a large multi-state outbreak (Mandrell, 2009) related to consumption of baby spinach was traced back to produce grown in central California coast (Jay et al., 2007). Indeed, EcO157 isolates that are genetically indistinguishable from the 2006 spinach outbreak strain were isolated from feral swine, cattle, surface water, and sediment samples near spinach fields (Jay et al., 2007). These results indicate wide-spread occurrence of this pathogen in numerous habitats, in the vicinity of produce production, each of which may affect survival of EcO157 differently. This produce growing region has been termed “The salad bowl of the US” since produce from this region are widely distributed to the entire US. Conversely, outbreaks have frequently been traced-back to the Salinas region. Therefore, environmental pathogens isolated from the Salinas region have the potential to impact public health throughout the US.

Although most EcO157 strains disappear rapidly from soil, manure and dairy wastewater environments (Avery et al., 2004; Berry and Miller, 2005; Ravva et al., 2006), a small proportion of cells remain viable for extended periods (Duffitt et al., 2011). Therefore, it is probable that most EcO157 cells within a population exposed to stressful environments outside the animal host fail to survive (Jones, 1999). Nevertheless, survival of some cells in water and soil results in transport of pathogen by surface water, irrigation and wind, possibly leading to produce contamination.

Survival of specific strains of EcO157 in sufficient numbers to cause infection is associated with their intrinsic fitness traits and genetic makeup (Topp et al., 2003). One such trait is the formation of biofilms, facilitated by the production of adhesins and exopolysaccharides (Cookson et al., 2002; Ryu and Beuchat, 2005). One such adhesin, curli, acts as a scaffolding agent in biofilms (Evans and Chapman, 2014) and is responsible for cell surface and cell to cell interactions (Reichhardt et al., 2015), immune activation, cell invasion and favor intestinal colonization (Saldaña et al., 2009; Biscola et al., 2011; Blanco et al., 2012; McWilliams and Torres, 2014). Curli are thin extra-cellular, proteinaceous aggregative fimbriae that act as a virulence factor by promoting attachment to eukaryotic cells (Uhlich et al., 2002; Kikuchi et al., 2005). Curli fimbriae, encoded on two divergently transcribed operons *csgDEFG* and *csgBAC*, are expressed in response to environmental stress factors such as low temperature, low oxygen, low osmolarity, and nutrient limitation (Barnhart and Chapman, 2006; Blanco et al., 2012).

Curli fibers are expressed at temperatures ranging from 25 to 37°C (Kikuchi et al., 2005; Barnhart and Chapman, 2006; Hung et al., 2014). Curli-expressing strains of EcO157 developed stronger association with leaf surfaces of produce (Patel et al., 2011; Fink et al., 2012; Macarisin et al., 2012) and *E. coli* strains isolated from plants appear to form significantly more curli compared to strains from humans or animals (Méric et al., 2013). In addition, curli producing strains significantly adhere to human epithelial cells compared to curli-deficient strain and form three-dimensional mature biofilms (Kikuchi et al., 2005). Biofilm formation by EcO157 isolates strongly correlated with curli production (Lee et al., 2011; Wang et al., 2012; Macarisin et al., 2014). Curli production in uropathogenic *E. coli* influences pathogenesis *in vivo* (Lim et al., 2014) and curli produced in the host provided a fitness advantage.

Subpopulations of EcO157 have been reported to adapt to harsh environmental conditions (Brzuszkiewicz et al., 2009) and environmental isolates appear to resist acidic conditions in comparison to clinical isolates from outbreaks (Oh et al., 2009). It is also known that curli-deficient variants of EcO157 survive better than curli producers under acidic conditions comparable to that of the stomach of the host (Carter et al., 2011). Curli-deficient strains and variant sub-populations persist longer in soil and resist protozoan predation compared to curli-expressing strains (Ravva et al., 2014a,b). Furthermore, the proportion of curli-expressing phenotype sub-populations of EcO157 decreased on repeated soil exposure while curli-deficient sub-populations increased. In fact, a curli-deficient strain of EcO157 was repeatedly isolated during a 45-day period from dry pasture soil indicating it's resilience and fitness in austere soil environment (Ravva et al., 2014a). In addition, the defense systems of certain plants like *Arabidopsis* recognize curli producing variants and adversely influence their survival and consequently curli-deficient EcO157 strain survive better on plants (Seo and Matthews, 2012). Thus, it is possible that environmental conditions aid in the enrichment of subpopulations with enhanced fitness.

Exposure to harsh environments may result in the development of stress resistant and/or virulent phenotypes. Our previous results indicated a possibility that soil exposure resulted in enriching a curli deficient subpopulation for survival (Ravva et al., 2014a). Consequently, it is likely other environmental conditions, particularly agricultural environments, also influence the survival of curli subpopulations. Here, we characterize phenotype differences in curli expression of 471 strains of EcO157 isolated from different environments (sample types) and associated with major outbreaks, sporadic infections and the fresh produce production environment of the central coast of California. These strains represent 328 phylogenetically distinct genotypes and were also compared to determine if genotypic differences influence the fitness selection of curli sub-populations.

## Materials and methods

### Strains of EcO157

EcO157 strains used in this investigation are from the culture collection of Produce Safety and Microbiology Research Unit of Western Regional Research Center located in Albany, California. Four-hundred and seventy one strains (Table 1 and Table S1) were selected based on sample source and genetic diversity determined by MLVA typing (Cooley et al., 2007). These strains representing 328 distinct MLVA types (Table S1) were associated with multi-state outbreaks, sporadic infections and a variety of environmental sources. Majority of the environmental strains were isolated from soil, water and sediment from farms and ranches in Monterey and/or San Benito counties in the central coast of California. Some of these farms were linked by traceback studies from produce-related outbreaks in Washington State in 2002 and in California during 2003 and a multi-state spinach outbreak in 2006. Detailed information on origin of strains and the environments they were isolated from is given in Table S1.

**Table 1:** **Summary of EcO157 strains characterized**.

**Isolate types**	**MLVA types**	**Number of strains (source^a^)**	**Total (%)**
2006 spinach outbreak	20	51(C), 2(W), 1(D) 13(P), 17(B), 11(F)	95 (20)
Other outbreaks	27	33(C), 4(F)	37 (8)
Sporadic incidences^c^	151	176(C)	176 (37)
Animal feces	82	48(B), 22(P), 15(O)	85(18)
Environmental isolates	65	24(S), 43(W), 11(D)	78(17)
Total	328^b^	260(C), 65(B), 35(P), 45(W), 15(O), 24(S), 12(D), 15(F)	471

a *Source codes: B, bovine; C, clinical; D, sediment; F, food; P, pig; S, soil; W, water; O, other animals*.

b *MLVA total is not additive with isolate types since identical MLVA types may occur in multiple isolates*.

c *Infections that occur infrequently and irregularly*.

### Genotype characterization

MLVA was performed using capillary electrophoresis methods described previously (Cooley et al., 2007). Essentially, 10 loci are amplified in three multiplex PCR reactions using fluorescent primers. All fluorescent primers were obtained from Applied Biosystems (ABI, Foster City, CA). Each PCR reaction of 10 μl contained 1X multiplex PCR master mix (Qiagen, Redwood City, CA), 0.2 mM of each primer and 1 μl template. Thermal cycling parameters for PCR reactions were 95°C for 15 min, then 25 cycles of 94°C for 30 s, 63°C for 90 s, 72°C for 90 s, with a final extension at 72°C for 10 min. The sizes of fragments were determined using ABI 3130xl Genetic Analyzer and GeneMapper software (ABI). Fragment sizes were converted to number of TR by subtraction of the amplified, non-repeat sequences and division by the repeat size. Fractional repeat numbers were rounded to the nearest whole repeat number for purposes of comparison. MLVA allele numbers were assigned according to published methods and with MLVA algorithms in Bionumerics software (Applied Maths, Sint-Martens-Latem, Belgium). MLVA allele number for each isolate tested in this study is listed in Supplemental Information, Table S1.

### Enumeration of phenotypic variant subpopulations

The proportion of phenotypic variants of EcO157 strains expressing curli was determined as described previously (Carter et al., 2011), with some modifications. Briefly, 10-fold serial dilutions of an isolated colony from overnight growth on LB agar, in 0.01 M PBS were plated on LB agar without NaCl, but supplemented with 40 μg/ml of Congo red dye (Sigma-Aldrich, St. Louis, MO) and 10 μg/ml of Coomassie brilliant blue G (Congo red agar, Sigma-Aldrich). Congo red dye binds to the cells without causing any growth inhibition (Reichhardt et al., 2015). Colony phenotype variants depend on the expression of curli and cellulose. Coexpression of curli and cellulose leads to red, dry and rough (*rdar*) colonies whereas inactivation of cellulose operon results in brown, dry and rough (*bdar*) colonies. Inactivation of curli operon results in pink phenotypes (*pdar*) and inactivation of both curli and cellulose genes generate smooth and white (*saw*) colonies (Uhlich et al., 2009). Colony phenotypes were determined after a 2 to 3-day incubation of plates at 28°C. In this study, both *rdar* and *bdar* colony phenotypes were counted as curli colonies and *pdar* and *saw* colony types were counted as curli-deficient colonies. Furthermore, EcO157 strains were grouped into four groups based on the proportion of curli phenotype variant colonies. These groups are 0–20% curli (curli-deficient), 21–50% curli, 51–80% curli, and 81–100% curli (predominantly curli-positive strains).

### Genotype and phenotype comparisons

Composite data sets comprising of variable number tandem repeat (VNTR) values and curli phenotype proportions for each strain as components were used to construct minimal spanning trees. Trees were constructed using the MST algorithm in the advanced cluster analysis of Bionumerics (Applied Maths). Manhattan distance function, coupled with UPGMA (unweighted pair group method with arithmetic mean) algorithm, was used in clustering VNTR values for the 11 loci and curli phenotype proportion of each strain. Similarity matrices of each were combined (VNTR data weighted 200:1) to form the composite data matrix for construction of the trees with N-locus variants weighted at 10,000 and 10 for *N* = 1 and *N* = 2, respectively.

### Statistical analysis

Comparison of curli expression among strains from branches of the minimal spanning tree used the statistical package in SigmaPlot version 13 (SyStat Software, Inc., San Jose, CA). Comparison of pairs of branches used Mann-Whitney Rank Sum Test. Larger analysis comparing multiple branches used Kruskal-Wallis One Way Analysis of Ranks. χ^2^ Analysis of Contingency Tables (SigmaPlot 13) was used to compare the distribution of curli expressing and non-curli expressing phenotypes from fecal and non-fecal environmental strains. Fisher Exact Test was used to compare the distribution of curli expression of non-fecal strains isolated from soil, water, and sediments.

## Results

### Strains

Shiga-toxigenic EcO157 strains isolated from infected patients during outbreaks and sporadic cases of infections that occurred during a span of 25 years (1982–2007; Table S1) were evaluated along with environmental isolates to determine if strains surviving various austere environments were phenotypically similar to those responsible for causing sporadic infections and illness outbreaks. All non-fecal and fecal isolates carry virulence (*stx*2, *eae, hly*) and serotype specific (*fli*C, *rfb*E) genes. Strains grouped into 328 genotypes based on 11-loci MLVA characteristics were evaluated to determine the relationship of illness-associated genotypes and the proportion of curli phenotypes. Twenty percent of strains were from the 2006 multi-state outbreak associated with the consumption of baby spinach (Table 1) and predominantly from a single MLVA genotype 163 (Table S1). Strains of the same genotype were isolated from infected individuals from all over the country and environmental samples from the vicinity of produce fields in central coast of California. More than one-third of all strains were from clinical sources of sporadic infections and another 8% were associated with other outbreaks. The characterized strains also include 85 and 78 strains isolated from a collection of animal and environmental sources, respectively.

**Figure 1:**
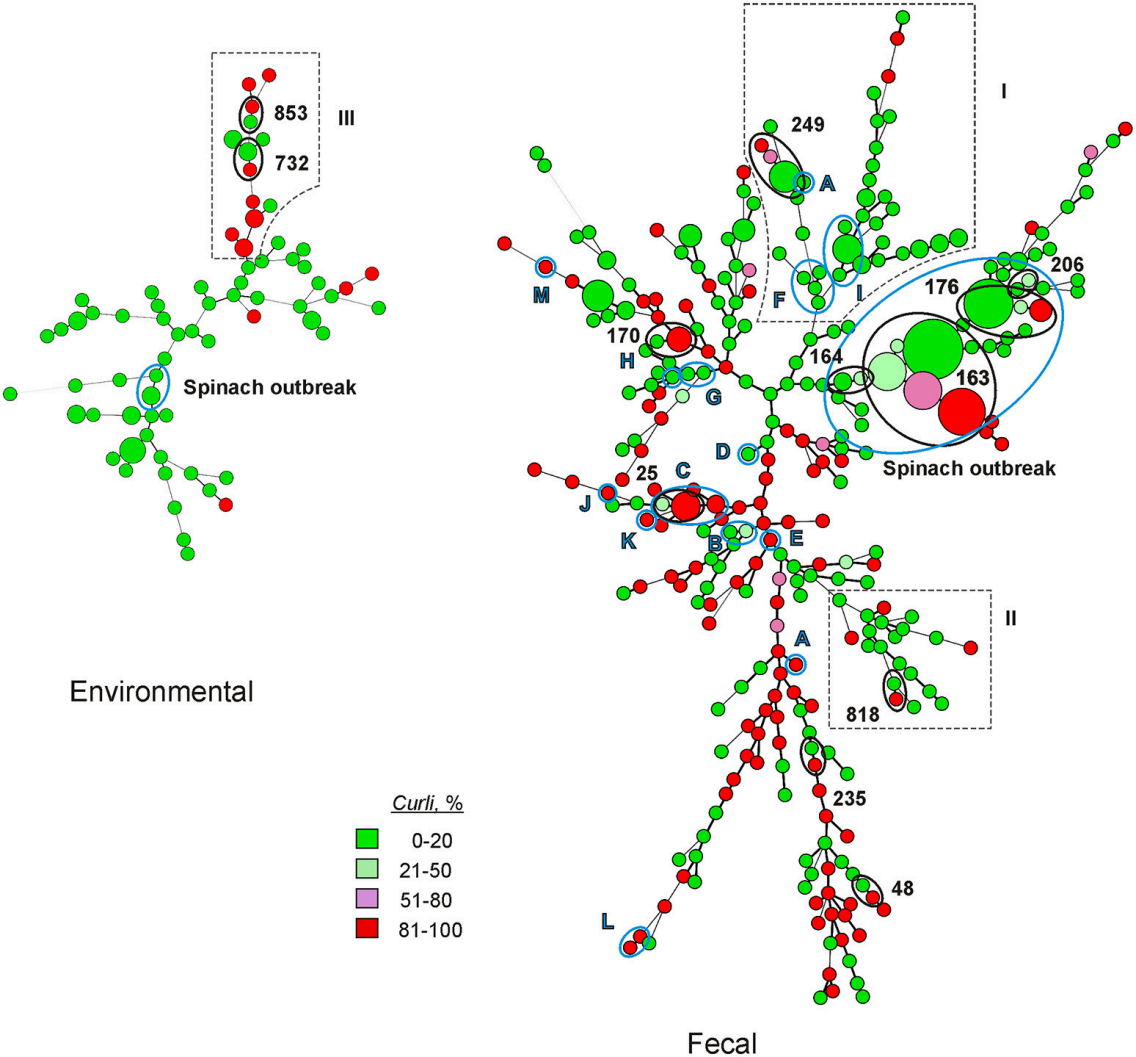
**Minimal spanning trees illustrating the phylogenetic relationship of fecal and environmental strains of EcO157**. EcO157 from outbreaks are indicated by blue circles and letters A–M in the trees. See Table 2 for outbreak labels. Spinach outbreak (2006) is labeled separately. Selected MLVA types (black circles and associated allele number) are also included in the trees showing the position of MLVA types with variant curli expression. Branches outlined with dotted lines (labeled I, II, and III) are branches with significant variant curli expression compared to other branches in the same tree. Size of circle represents the number of strains characterized for each MLVA genotype. Smallest circle denotes a single strain, while the largest circle represents 22 strains that are genetically indistinguishable from the 2006 spinach outbreak strain of MLVA genotype 163.

Strains from 328 MLVA genotypes were characterized to determine the disposition of the curli character as it relates to MLVA and sample type. For this purpose, environmental is restricted to inanimate substrates, such as soil, sediment, and water (Figure 1). Animals surveyed in these experiments were part of farm and ranch environments, but feces, be it human or animal, offers a very hospitable environment to enteric bacteria, very different from the harsh environment of soil, water and sediment. As such, animal fecal isolates were analyzed with human fecal isolates associated with outbreak and sporadic infections. Of all the strains characterized, 64% were curli-deficient and 30% were curli-positive. Only a small fraction (6%) of strains had phenotype proportions ranging between 20 and 80% curli.

### Curli expression by fecal/outbreak strains of EcO157

Curli expression of fecal strains from outbreaks, sporadic infections and domestic and wild animals is shown in Table 2. More than half of clinical strains in this study did not express curli. EcO157 strains from several outbreaks linked with the consumption of contaminated apple juice, produce, hamburgers, steak and beef were also curli-deficient phenotypes. However, in these cases (Table 2, D to I) only a few strains from each outbreak were available for testing. In several outbreaks where 8 or more strains were available curli was expressed to varying degrees. The exceptions were the Romaine leaf and Pat & Oscar outbreaks which showed varying curli expression with only two strains tested (Table 2). Nevertheless, with the two largest collections of strains, Spinach outbreak 2006 and the sporadic infection, the expression of curli was 27 or 42%, respectively, with a small number of strains showing intermediate levels of expression. Likewise, strains from animal feces showed curli expression where more than a few strains were analyzed, i.e., with cattle and feral pig feces the level of expression was 25 and 9%, respectively. A significant proportion (*P* < 0.001) of animal fecal isolates (84%) gave no curli expression compared to human fecal isolates (58%). Incidentally, ConAgra ground beef outbreak (2002) strains (Figure 1, Table 2) were indistinguishable from the spinach outbreak strain of MLVA 163 by PFGE.

**Table 2:** **Curli phenotype proportion of fecal strains of EcO157 linked with sporadic infections, outbreaks and domestic and wild animals**.

**Strains from feces**	**No. of strains**	**Percent strains in each curli group**				**Tree Label^a^**
		**0–20%**	**21–50%**	**51–80%**	**81–100%**	
All fecal strains	375	63	3	3	31	
Spinach outbreak 2006^b^	51	57	8	8	27	
Sporadic infections^c^	176	54	1	3	42	
Romaine lettuce outbreak 2002	2^d^	50	0	0	50	A
Pat & Oscars outbreak 2003	2^d^	50	50	0	0	B
Taco bell outbreak1999	8	12	12	0	75	C
Western states outbreak 1993	1	100	0	0	0	D
Odwalla outbreak 1996	1	100	0	0	0	E
Iceberg lettuce outbreak 1999	4	100	0	0	0	F
ConAgra outbreak 2002^e^	2	100	0	0	0	G
Taco bell outbreak 2006	1	100	0	0	0	H
Peppa's outbreak 2010	7	100	0	0	0	I
Walkerton outbreak 2000	1	0	0	0	100	J
Spinach outbreak 2003	1	0	0	0	100	K
TGIF outbreak 2006	2	0	0	0	100	L
Hamburger outbreak 2000	1	0	0	0	100	M
Bovine feces	65	71	5	2	23	
Feral pig feces	35	91	0	0	9	
Cowbird feces	4	100	0	0	0	
Coyote feces	3	100	0	0	0	
Crow feces	5	100	0	0	0	
Kangaroo rat feces	1	100	0	0	0	
Elk feces	1	100	0	0	0	
Oregon junco	1	100	0	0	0	

a *Outbreak labels for nodes in the minimal spanning trees in Figure 1. All outbreak strains are clinical isolates*.

b *Clinical isolates from the spinach outbreak 2006*.

c *Infections that occur infrequently and irregularly*.

d *Both strains are of different MLVA genotypes*.

e *PFGE patterns indistinguishable from those of the spinach outbreak strain*.

### Curli expression in strains from non-fecal sources

Non-fecal strains, representing 20% of strains evaluated, were mostly curli-deficient (71%, Table 3) and were isolated from soil, water, sediment and food. The distribution of curli phenotypes from soil did not differ significantly from the strains from water (*P* = 0.454) or sediment (*P* = 0.664). Excluding strains from food, 82% of the environmental strains failed to express curli and were similar in curli expression by animal fecal strains (84% curli negative; *P* = 0.558). Compared to human fecal isolates (58%), a significant proportion of non-fecal strains (80%) gave no curli expression (*P* < 0.001). Curli composition of isolates from spinach bags were unique in this group, in that, a high proportion of strains exhibited both curli-deficient and curli-positive clonal sub-populations.

**Table 3:** **Curli phenotype distribution of non-fecal strains of EcO157**.

**Source**	**No. of strains**	**Percent strains in each curli group**			
		**0–20%**	**21–50%**	**51–80%**	**81–100%**
Total	96	71	5	5	19
Sediment	12	75	5	0	25
Soil	24	83	0	0	17
Water	45	82	0	0	18
Spinach bag	11	9	45	45	0
Apple Juice- Odwalla outbreak^a^	1	100	0	0	0
Hamburger–Emmpak Foods outbreak^a^	1	0	0	0	100
Lettuce–Taco Johns outbreak^a^	2	50	0	0	50

a *Isolated from food and are not clinical isolates*.

### Phylogenetic analysis of curli expression

Minimal spanning trees were constructed to include both VNTR values and curli proportions in percentage groups as above. The disposition of curli expression within the Fecal tree was nearly evenly dispersed. No branches contained exclusively curli expressing or curli non-expressing strains. Nevertheless, two major branches in the Fecal tree (labeled I and II in Figure 1) contained strains with significantly reduced curli expression (*P* < 0.05) compared to other branches in that tree. Those strains showing differential expression of curli from several outbreaks were clustered and several of these showed intermediate levels of expression, as indicated. Several of these clusters involve strains with identical MLVA types from multiple sample sources with varying levels of curli expression. Where this occurs is noted by indicating the MLVA allele and the source of the strains involved. A separate tree was constructed exclusively from environmental strains since it was noted in Table 3 of a large predominance of non-curli expressing strains. Most curli expression in this tree is limited to a single branch (labeled with III in Figure 1) that is significantly different compared to other branches in the tree (*P* < 0.01). This branch of predominantly expressing strains does not include strains from the 2006 spinach outbreak, but does primarily include water, sediment and soil isolates from only an 11 month period of time in 2009 and 2010 and sampled within a restricted portion of the Salinas valley. Soil and sediment isolates recovered from this region were sampled from cattle ranches or streams closely situated to the same ranches. In this branch also noted are two examples of strains with identical MLVA types showing differential expression of curli.

## Discussion

Predicting the likely occurrence of foodborne outbreaks linked with the consumption of fruit and vegetable produce is extremely important but difficult in practice, as the survival, proliferation, and transport of the pathogen are all controlled by both multiple environmental and biological factors. Thus, survival of EcO157 has been reported to vary from nearly 2 years in manure piles (Kudva et al., 1998) to less than a day in dairy wastewater (Ravva et al., 2006). Likewise, it is intriguing that even a strain that survived on apples and in apple juice in sufficient number to cause an outbreak in 1996 also rapidly disappeared in a day in manure wastewater. These results indicate that EcO157 strains respond differently to different environmental conditions. However, strain specific factors are also important in fitness of EcO157 in human and animal host and in the environment. Strain specific differences in curli (Parker et al., 2012), biofilm formation (Wang et al., 2012), and shiga-toxin expression (Neupane et al., 2011) influence environmental fitness and may contribute to the likelihood of an outbreak.

Fecal isolates showed a uniform distribution of curli expression (Figure 1, Table 2). This situation was especially noticeable where multiple fecal strains from the same outbreak were analyzed. Some outbreaks associated with predominantly curli-deficient strains were caused by the consumption of contaminated water (2000), apple juice (Odwalla outbreak of 1996), lettuce (1999, 2002, and 2006), and ground beef (2002). Yet, in these cases, the sampling of available strains from each outbreak was rather small, limiting the conclusions that can be made. Significantly, the large collection of 2006 spinach outbreak strains showed nearly 50% curli expression. Likewise, the large collection of unrelated sporadic strains gave a similar result, supporting the suggestion that curli expression in these strains is more dependent on sample source than on genotype. Curli expression in bovine and feral pig fecal strains similarly showed a mixture of curli deficient and curli prevalent phenotypes, though the curli deficient group was at a significantly higher percentage, compared to human fecal strains. It is noteworthy, that the sampling methods for animals and humans were substantially different (unpublished results). In many cases, animal fecal samples were collected from the ground and, as such, were exposed to the environment for a period of time and may have experienced considerable stress. Consequently, these strains may be substantially more “environmental” than other fecal isolates collected directly from the gut.

Although curli fimbriae promote adherence to surfaces (Barnhart and Chapman, 2006) and help to colonize animal tissues (Uhlich et al., 2002; Rosser et al., 2008), it is remarkable to find more than half of clinical strains from outbreaks and sporadic cases of infections are curli-deficient. Nevertheless, it is possible that curli plays a critical role in biofilm formation in different environments (Saldaña et al., 2009; Wang et al., 2012). For instance, curli-deficient variants are known to be acid-resistant (Carter et al., 2011) and it is likely that the acid sensitive curli-producers are eliminated in the stomach of the animal host. Thus, the results presented here indicate that a lower percentage of curli expressing strains are found in animal feces and environmental samples. Consequently, selection for curli expressing strains may occur in the gut of cattle and humans, and perhaps, to a lesser degree in other species. Additionally, some plants appear to recognize curli and induce defense systems. Hence, pathogens expressing curli survive poorly on plant surfaces (Seo and Matthews, 2012). However, *csg*A (a gene encoding curli biosynthesis) mutants of an EcO157 strain showed no reduction in their ability to bind to alfalfa sprouts, suggesting that the pathogen may have more than one mechanism for binding to plant surfaces (Jeter and Matthysse, 2005). These results indicate that curli is part of a collection of various fitness capabilities selected to survive multiple, stressful environments.

Except for some strains from bagged-spinach and food isolates, 82% of environmental strains are curli-deficient (Figure 1, Table 3). The prevalence of curli-deficient strains from the environment was predicted as some curli-deficient strains survived longer in soils and some of the same strains also resisted predation by protozoa in dairy waste water (Ravva et al., 2014a,b). Furthermore, curli-deficient clonal sub-populations of both clinical and environmental strains survived longer in soil compared to the curli-positive clones of the same strains (Ravva et al., 2014a). These conclusions are further supported here with additional strains from soil, but also strains from water and sediment (Figure 1, Table 3). Thus, curli-deficient strains and curli-deficient sub-populations have fitness traits for survival in harsh produce production environments.

We observed intra-genotype differences in proportion of curli phenotype expression, possibly based on environmental exposure or residence in human or animal host. The 2006 spinach outbreak strains, indistinguishable from each other by MLVA genotyping, differ substantially in phenotypic expression. Half of the clinical strains of MLVA type 163 failed to express curli while isolates of the same genotype from cow feces exhibited phenotypes ranging from no expression of curli to 100% clones that are curli (Figure 1, Table S1). Several other MLVA genotypes showed variation in curli expression, but these genotypes failed to cluster, possibly indicating that variation of curli expression is not linked to specific genotypes. However, one branch of the minimal spanning tree of environmental strains (Figure 1) showed preferential curli expression. It is also noteworthy that the sample locations of 8 of these strains were within a 31 km region in Salinas and during a 4 month period (data not shown). Consequently, their close phylogenetic and temporal/spatial relationship may indicate transport and variation of a strain from a single point in the environment. Importantly, this instance of potential genetic control of curli may be only apparent within the background of relatively invariant environmental isolates. Since, the majority of fecal strains show variable curli expression, the genotype effects may have been masked by influence of the environment in the gut. Although it needs further validation using many more genotypes, from this same cluster and others, one can predict that certain genotypes may preferentially express curli.

In summary, these results expand on previous observations contrasting curli expression in fecal and more austere environmental isolates, to include a variety of domestic and wild animal fecal strains and a large group of water, sediment, and soil isolates from a major agricultural region of California. We have demonstrated that curli expression of clonal subpopulations depends primarily upon the type of environmental exposure and the isolation source, although genetic differences also contribute somewhat to clonal variation in curli expression. Further experiments will be necessary to quantify these effects as it relates to degree and duration of exposure to these environments. Nevertheless, the curli-deficient phenotype may be a selective trait for survival and proliferation in austere produce production environments.

## Author contributions

SR designed and conducted experiments, analyzed, and wrote the manuscript. CS and MC conducted experiments, analyzed, and contributed to the manuscript preparation.

### Conflict of interest statement

The authors declare that the research was conducted in the absence of any commercial or financial relationships that could be construed as a potential conflict of interest.

## Supplementary material

Table S1 **EcO157 strains used in this study**. Details include MLVA genotype, percent curli subpopulations, sample source, and year of isolation.
